# Examining use of restaurant nutrition information among adults living in England engaged in disordered eating or weight management efforts

**DOI:** 10.1186/s12889-026-27141-6

**Published:** 2026-03-31

**Authors:** Alena F. Oxenham, Amanda Raffoul, Christine M. White, Lana Vanderlee, David Hammond, Michael Essman

**Affiliations:** 1 MRC Epidemiology Unit, School of Clinical Medicine, University of Cambridge, Cambridge, UK; 2 Mohn Centre for Children’s Health and Wellbeing, School of Public Health, Imperial College London, London, UK; 3 Department of Nutritional Sciences, University of Toronto, Toronto, ON Canada; 4 School of Public Health Sciences, University of Waterloo, Waterloo, ON Canada; 5 École de nutrition, Centre Nutrition, santé et société (NUTRISS), Université Laval, Québec, Québec Canada; 6 Sanford School of Public Policy, World Food Policy Center, Duke University, Durham, NC USA

**Keywords:** Food policy, Menu labels, Disordered eating, Weight management

## Abstract

**Background:**

Calorie labels on menus are a popular policy initiative to help consumers to reduce their energy intake. Consumers trying to improve their diet may benefit from the labels if they help inform eating decisions. However, it is not well known how some groups, such as those with disordered eating, may view a calorie labelling policy and engage with nutritional labels in England.

**Methods:**

Using 2021 International Food Policy Study data from 2,898 adults in England, we assessed policy support for menu labels, participants’ noticing and influence of nutrition information, and behavioural impacts based on nutrition information labelling at restaurants. Using logistic regression models, we explored whether there were differences in responses between participants engaged in disordered eating or weight management efforts compared to those who engaged in neither.

**Results:**

A majority (56.9%) of participants supported a calorie labelling policy. People reporting disordered eating behaviours had greater odds of noticing, being influenced by, and changing behaviour because of nutritional information compared with participants not engaging in disordered eating or weight management. Participants with a preoccupation with thinness had greater odds of supporting calorie labelling, whereas those engaging in binge eating did not differ in odds and those reporting self-induced vomiting had lower odds of supporting the policy. Compared to the reference group, weight loss and weight maintenance groups had greater odds of being influenced by nutrition information and of eating out less often. Of the weight management groups, only participants reporting weight loss efforts had greater odds of ordering something different and of supporting a calorie labelling policy.

**Conclusion:**

As one of the first quantitative studies in England to examine engagement with restaurant nutrition information among adults with disordered eating and weight management efforts, this study indicates that nutrition information had differential impacts among those with disordered eating or engaging in weight management. England implemented a mandatory calorie labelling policy for the out-of-home food sector after the current data were collected; future studies should examine how the policy influences eating decisions and consider potential impacts on people with disordered eating.

**Supplementary Information:**

The online version contains supplementary material available at 10.1186/s12889-026-27141-6.

## Introduction

The out-of-home food sector is diverse, but often characterised by energy dense foods, and greater consumption of out-of-home food has typically been associated with lower diet quality [1–4]. Eating out-of-home is increasingly prevalent in diets globally and is predicted to increase over time [5]. In the UK, reports from 2015 to 2019 revealed that over 25% of adults ate outside their homes at least once a week [6, 7]. While there was a decline in eating out of home following the onset of the COVID-19 pandemic, the prevalence increased by approximately 10% from 2022 to 2023 [8].

Previous work has shown that consumers struggle to accurately estimate their caloric intake when eating [9]. Research from the United States has shown that individuals may not be aware of their daily caloric needs and face challenges in determining the energy content of foods, particularly in fast-food settings [9, 10]. In England, customers at out-of-home food outlets have been found to underestimate calories purchased by an average of 253 kcal [11]. One potential policy response to help mitigate these issues is the implementation of calorie labels in the out-of-home food sector. By presenting energy and nutrient information on menus, these labels aim to empower consumers to make informed choices about their caloric intake and incentivize businesses to offer lower caloric options [12, 13].

### Calorie labelling policies as a public health initiative

Calorie labelling policies may be seen as a nutrition-related policy option that is favourable for governments because it is minimally invasive on personal liberties and intervenes at the level of individual decision-making instead of restricting choices for the consumer. From a public perspective, government interventions are generally more accepted if they are perceived as less intrusive, even though research from other health-related fields indicates informational interventions generally require higher levels of agency and individual action and are often less effective at changing behaviours (e.g. smoking or alcohol consumption) than more restrictive policies [14, 15]. In particular, research has shown that those interested in managing their weight may be more likely to notice nutrition information and stand to benefit the most from a calorie labelling policy [16–18].

### Menu labels have mixed effects on calorie consumption

There have been mixed reports of the effectiveness of calorie labelling policies on reducing caloric intake. A previous meta-analysis found no overall significant effects of menu labels on calories ordered or consumed in studies conducted in a mix of restaurant and laboratory settings, although the studies specifically conducted in laboratory settings did show a reduction in calories ordered or consumed [19]. Similarly, two other reviews found some small but significant reductions in calories ordered with menu labels in laboratory settings, but found that this effect did not persist in studies conducted in restaurant or real-world settings [17, 20]. Another meta-analysis showed that overall, menu labels were associated with reductions in calorie consumption and ordering, but that the effect was smaller, although still significant, in real-world settings [21].

### Potential impact of calorie labelling policies on those with disordered eating

Although calorie labels have higher support among the general public than more stringent policies [14, 15], levels of acceptance may vary among subpopulations. For instance, concern has been expressed by some advocacy groups about potentially harmful effects of a calorie labelling policy on people living with disordered eating [22]. Disordered eating is defined by a range of attitudinal and behavioural characteristics, including preoccupation with thinness and binge eating, and is categorized by unhealthy and extreme weight control behaviours, such as using diet pills, diuretics, or self-induced vomiting [23, 24]. Disordered eating is characterised by unhealthy eating behaviours and attitudes, but may not meet the full criteria for a clinical eating disorder diagnosis [24]. It is also linked to a higher risk of adverse outcomes, including weight gain and eating disorders​ [23]​.

A 2007 UK-based survey found a 6.3% of respondents displayed disordered eating patterns [25]; a similar prevalence of 10.1% was found in a South London population of adults older than 16 years ​ [26]. A longitudinal study from 2017 found that over half of adolescents in a UK cohort reported at least one symptom of compensatory behaviours, including purging, hiding or throwing away food, and skipping meals [27]. The prevalence of disordered eating has been more extensively explored among youth and young adults than the general adult population, with higher rates of disordered eating being found in these groups [23, 28–30]. The 2019 Global Burden of Disease Study found a 15% increase in prevalence of eating disorders from 1990 to 2019 among young European populations [31]. Given the relatively high prevalence of disordered eating, it is vital to consider how a calorie labelling policy may impact these populations.

There are mixed empirical findings on the relationship between calorie labels and disordered eating. A recent systematic review and meta synthesis, primarily consisting of qualitative studies found that people with disordered eating are more likely notice nutrition labels and change their behaviour due to the labels, and identified themes of being drawn to calories and using labels to facilitate disordered eating [32]. Qualitative and mixed-method studies have shown that some people experience negative emotions related to food and eating when interacting with calorie labels, especially those with high disordered eating scores [33, 34]. People living with disordered eating have reported that calorie labels add tension or strain to their relationships when eating out, or that they avoid restaurants because of calorie labels and increased feelings of shame and isolation [35, 36].​ A hypothetical online task including female undergraduate students with disordered eating found the impact of calorie labels varied by the type of eating disorder [36]. Other research did not find an interaction between disordered eating and calorie labels affecting consumption [37, 38].

Due to the mixed results of studies examining the impacts of calorie labelling on those with and without disordered eating, further research is required to address the knowledge gap of how people in England with disordered eating notice and use menu labelling, as well as whether this population supports or opposes the menu labelling policy. The aim of the current study was to examine the prevalence of noticing, using, and support for nutrition information on menus at restaurants among different subgroups of adults in England including those engaged in disordered eating, attempting to manage their weight, and neither.

## Methods

### Dataset

Data were collected through a self-reported online survey as part of the UK arm of the 2021 adult International Food Policy Study (IFPS) in November and December 2021 (prior to the implementation of mandatory calorie labelling in England). Participants were recruited through the Nielsen Consumer Insights Global Panel and their partner’s panels. Random samples were drawn with quotas for age group and sex to approximate the known proportions in the UK. Eligible participants were current residents in the UK between the ages of 18 and 100. This analysis was restricted to participants from England, as the menu labelling policy has not yet been announced in other areas of the United Kingdom. All participants provided informed consent before participating in the online survey. Participants were given rewards according to their panel’s usual incentive structure. The study was reviewed by and received ethics clearance through a University of Waterloo Research Ethics Board (REB# 30829). Further survey methodology information can be found in the IFPS Technical Report [39]. Data were collected from 4,196 participants in the 2021 UK IFPS Survey. Among those, 622 (14.8%) respondents who did not reside in England, and 46 (1.1%) respondents with missing ethnicity, sex, income, or education data were excluded from the analytic sample. An additional 630 participants (15.0%) were excluded for not having visited a restaurant in the past 6 months, since several questions of interest were only asked to respondents who had visited in the last 6 months. The final analytic sample size was 2,898.

### Exposure groups

#### Disordered eating (DE) groups

The IFPS survey assessed potential characteristics of disordered eating using a 3-item behavioural and attitudinal tool derived from the Eating Attitudes Test (EAT-26), which screens for risk of eating disorders and has been assessed for validity among U.S. adolescents (Haines et al.). The screener includes two behavioural and one attitudinal item, the combination of which was found to be more sensitive than using attitudinal measures alone [40]. Participants were asked about their binge eating and vomiting behaviours over the past three months, as well as their preoccupation with a desire to be thinner (*Supplementary Table 2*). Cut offs for each of the three items were set as follows: going on an eating binge once or more a month; vomiting to control weight once or more a month; and often, usually, or always preoccupied with thinness. Unlike the original validation study, the same cut off for preoccupation with thinness were used for both men and women, as to not reinforce the narrative that women have a higher threshold for preoccupation with thinness than men. Based on these cut-offs, 779 participants were classified as in the binge eating group, 530 in the self-induced vomiting group, and 832 participants as preoccupied with thinness (*Supplementary Table 3*).

#### Weight management with no disordered eating groups

Respondents were asked to indicate whether they have tried to manage their weight: “During the past 12 months, have you tried to… lose weight, gain weight, stay the same weight, I have not tried to do anything about my weight” (Supplementary Table 2). Participants who reported weight management efforts and did not report any disordered eating behaviours or attitudes were coded as their respective weight management behaviour groups. In total, 83 participants were classified as in the weight gain effort group, 581 in the weight loss efforts group, and 387 in the weight maintenance efforts group (Supplementary Table 3).

#### Reference group

Participants who did not report any disordered eating or weight management efforts were classified as the “reference group”. 624 participants were included in this group. The exposure groups and their classifications are summarized in Supplementary Table 2 in the Appendix.

### Outcome measures

The main outcomes of interest were noticing and influence of nutrition information at last restaurant visit, impact of nutrition information in restaurants over the past six months, and policy support of calorie labels on chain restaurant menus. The noticing and influence items asked about participants’ last restaurant visit, whereas the impact items ask about the last 6 months, referring to usual behaviours. Survey questions, response options, and variable coding are described in Table 1.

**Table 1:** Primary outcome variables classifications

Variable	Question	Response Options	Coding
Policy Support	“Would you support or oppose a government policy that would require calorie amounts on menus of chain restaurants?”	Support, Neutral, Oppose, Don’t know, Refuse to answer.	Support No Support (Neutral, Oppose) *Missing (DK/R)*
Noticing of Nutrition Information	“The last time you visited a restaurant, did you notice any nutrition information?”	Yes, No, Don’t know, or Refuse to answer.	Noticed (Yes) Did Not Notice (No) *Missing (DK/R)*
Influence of Nutrition Information (if Noticing of Nutrition Information = yes)	“Did the nutrition information influence what you ordered?”	Yes, No, Don’t know, Refuse to answer	Influenced (Yes) Not Influenced (No) *Missing (DK/R)*
Impact of Nutrition Information (Behaviours)	“In the past 6 months, have you done any of the following because of nutrition information in restaurants?”	Ordered something different Eaten less of the food you ordered Changed which restaurants you visit Eaten at restaurants less often	Yes (selected) No (did notselect) *Missing (DK/R)*

### Sociodemographic characteristics

Sociodemographic characteristics were chosen as covariates due to their associations with menu labelling noticing and use, as established in the literature [17]. Sociodemographic characteristics assessed included age (continuous), sex assigned at birth (male or female), gender, highest education completed (secondary school education or less, or higher education), ethnicity, and perceived income adequacy [41]. A description of the sociodemographic variables and their categorizations can be found in *Supplementary Table 4*. Sex assigned at birth was chosen as a covariate instead of gender identity due to the very small sample sizes in gender categories other than cisgender “Man” or “Woman” that precluded the inclusion of these individuals in the analysis. Gender identity is included as a descriptive characteristic in Table 2.

### Statistical analysis

Statistical analyses were conducted using Stata, version 17 [42]. All analyses were weighted using post-stratification sample weights constructed using a raking algorithm with country-specific population estimates based on age, sex, region, ethnicity, and education [39].

Descriptive statistics were used to summarize the sociodemographic characteristics of the analytic sample, including means (SD) or n (%) as appropriate.

#### Logistic regression analyses

Separate logistic regression models were run for each outcome (menu label policy support, noticing and influence of nutrition information, and the four behavioural impact items) to explore whether there were differences in outcomes across the individual six exposures (binge eating, self-induced vomiting, preoccupation with thinness, weight gain with no disordered eating, weight loss with no disordered eating, and weight maintenance with no disordered eating) compared to the reference groups (no disordered eating and no weight management) in order to examine differences in outcomes between the different disordered eating and weight management groups. Models were adjusted for age, sex, ethnicity, education, and perceived income adequacy. The results are reported as odds ratio (OR) and 95% confidence intervals (CI) for all outcomes. Individual models were chosen instead of a joint model because of the potential overlap between the subgroups of disordered and weight management (e.g. participants could have reported multiple disordered eating or weight management behaviours), and to keep the reference group consistent for each subgroup. In a joint model, each exposure would be compared against “everyone without that exposure,” including individuals with other disordered eating or weight management behaviours, which could obscure subgroup-specific associations. This approach prioritised interpretability of subgroup-specific associations over model simplicity. Due to the large number of statistical tests (42 in total) run in this subgroup analysis, the Benjamini-Hochberg procedure was used to control for false discovery rates [43, 44]. The false discovery rate was set to 0.05.

## Results

Descriptive results are reported in Tables 2 and 3 as the weighted number of participants (n%), rounded to the nearest integer. Prevalences of each exposure group and cross-tabulations between reports of weight management and disordered eating are found in Supplementary Tables 3 and 4 in the Appendix. The demographic distribution of the exposure groups can be found in Supplementary Table 4.

**Table 2:** Sample demographic characteristics (weighted n; weighted %) (*n* = 2,898)

Characteristic	Total Weighted *n* (weighted %)
Sex (assigned at birth)	
Male	1405 (48.5)
Female	1493 (51.5)
Gender	
Man	1385 (47.8)
Woman	1492 (51.5
Trans male/trans man	5 (0.2)
Trans female/trans woman	4 (0.1)
Gender queer/nonconforming	8 (0.3)
Different identity	4 (0.2)
Age	
Mean (SD)	48 (17.3)
Ethnicity	
White	2507 (86.5)
Multiple or other ethnic groups	138 (4.8)
Asian/Asian British	182 (6.3)
Black/African/Caribbean/Black British	72 (2.5)
Income Adequacy:	
Not easy	1636 (56.5)
Easy	1262 (43.6)
Highest Education	
Secondary school or lower	2022 (69.8)
Post-secondary school or higher	876 (30.2)

**Table 3:** Weighted n (%) of the population for each outcome (*n* = 2,898)

	Yes*n* (%)	No*n* (%)	Don’t Know/Refuse/NA*n* (%)
Noticed nutrition information at last restaurant visit	544 (18.8)	2266 (78.2)	88 (3.0)
Influence of nutrition information at last restaurant visit*	316 (10.9)	225 (7.8)	2356 (81.3) *
Ordered something different in response to nutrition information in past 6 months	409 (14.1)	2489 (85.8)	0 (0.0)
Ate less of food ordered in response to nutrition information in past 6 months	373 (12.9)	2525 (87.1)	0 (0.0)
Ate out less often in response to nutrition information in past 6 months	279 (9.6)	2619 (90.4)	0 (0.0)
Changed restaurants in response to nutrition information in past 6 months	291 (10.0)	2607 (90.0)	0 (0.0)
Policy Support (Support vs. Non-Support)	1649 (56.9)	1193 (41.2)	56 (1.9)

*The influence item was only asked of respondents who answered “yes” to the previous noticed nutrition information item

### Differences in menu labelling outcomes between sub-groups

All three of the disordered eating groups had greater odds of reporting noticing nutrition information in the last 6 months compared to the no weight management and no disordered eating reference group (Table 4). Odds of noticing did not differ for participants who reported trying to gain, lose, or maintain their weight compared to the reference group (Table 5).

**Table 4:** Prevalence (%) and adjusted ORs and 95% CIs of the associations between disordered eating exposure groups and outcomes

Outcome	Binge eating (*n* = 779)	Self-induced vomiting (*n* = 530)	Preoccupied with thinness (*n* = 832)	Reference group (No weight management efforts, no DE) (*n* = 624)
Noticed nutrition information at last restaurant visit?	32.3% 3.24 (2.08–5.04) p = 0.017*	45.8% 5.55 (3.37–9.14) p = 0.001*	29.9% 2.90 (1.96–4.29) p = 0.014*	*10.4% * *ref*
Influenced by nutrition information at last visit?	25.0% 20.68 (7.33–58.34) p = 0.008*	37.0% 20.36 (7.11–58.30) p = 0.011*	21.6% 13.38 (4.91–36.41) p = 0.019*	*1.5% * *ref*
In the past 6 months, have you done any of the following because of nutrition information in restaurants?				
Ordered something different	22.8% 4.51 (2.68–7.61) p = 0.007*	28.2% 7.52 (3.80-14.88) p = 0.006*	24.0% 4.72 (2.96–7.53) p = 0.002*	*5.7%* *ref*
Ate less of food ordered	29.4% 6.53 (3.29–12.96) p = 0.013*	37.2% 8.83 (4.33–17.99) p = 0.005*	24.0% 5.74 (2.90-11.36) p = 0.018*	*3.3% * *ref*
Ate out less often	13.9% 3.76 (2.14–6.60) p = 0.021*	15.1% 4.48 (2.15–9.36) p = 0.026*	10.7% 2.58 (1.55–4.33) p = 0.030*	*3.9% * *ref*
Changed restaurants	23.5% 8.56 (3.91–18.74) p = 0.012*	33.4% 18.65 (7.21–48.23) p = 0.004*	18.9% 7.58 (3.56–16.12) p = 0.015*	*2.0% * *ref*
Support a calorie label policy?	47.6% 1.28 (0.94–1.74) *p* = 0.045	37.9% 0.66 (0.46–0.95) p = 0.043*	60.7% 2.01 (1.52–2.66) p = 0.020*	*52.0* *ref*

*Significance is represented by an * for each *p*-value that was below the critical value derived from the Benjamini-Hochberg calculations

**Table 5:** Prevalence and adjusted ORs and 95% CIs of the associations between weight management exposure groups and outcomes

Outcome	Weight Gain Efforts, no DE (*n* = 83)	Weight Loss Efforts, no DE (*n* = 581)	Weight Maintenance Efforts, no DE (*n* = 387)	Reference group (No weight management efforts, no DE) (*n* = 624)
Noticed nutrition information at last restaurant visit?	11.8% 0.76 (0.31–1.84) *p* = 0.543	10.3% 0.99 (0.63–1.54) *p* = 0.952	12.1% 1.17 (0.75–1.83) *p* = 0.488	*10.4%* *ref*
Influenced by nutrition information at last visit?	4.9% 3.21 (0.53–19.52) *p* = 0.202	4.1% 5.80 (1.99–16.88) p = 0.001*	5.4% 5.46 (1.70-17.51) p = 0.005*	*1.5% * *ref*
In the past 6 months, have you done any of the following because of nutrition information in restaurants?				
Ordered something different	15.2% 2.27 (1.01–5.10) *p* = 0.047	10.2% 1.79 (1.09–2.94) p = 0.021*	7.5% 1.28 (0.72–2.28) *p* = 0.391	*5.6%* *ref*
Ate less of food ordered	11.8% 1.36 (0.49–3.79) *p* = 0.559	5.5% 1.68 (0.79–3.58) *p* = 0.179	3.6% 0.92 (0.40–2.11) *p* = 0.837	*3.3% * *ref*
Ate out less often	10.0% 2.37 (0.74–7.62) *p* = 0.147	9.8% 2.69 (1.61–4.52) p < 0.001*	10.7% 2.83 (1.65–4.87) p < 0.001*	*3.9%* *ref*
Changed restaurants	10.4% 2.07 (0.44–9.66) *p* = 0.353	2.9% 1.70 (0.70–4.15) *p* = 0.243	4.5% 1.97 (0.82–4.72) *p* = 0.130	*2.0%* *ref*
Support a calorie label policy?	59.1% 1.48 (0.82–2.65) *p* = 0.193	68.7% 1.97 (1.49–2.59) p < 0.001*	58.9% 1.31 (0.98–1.76) *p* = 0.067	*52.0%* *ref*

*Significance is represented by an * for each *p*-value that was below the critical value derived from the Benjamini-Hochberg calculations

Among the participants who reported noticing nutrition information, all three of the disordered eating groups had greater odds of reporting being influenced by the information in the past 6 months compared to the no weight management and no disordered eating reference group (Table 4). Compared to the reference group, the odds of being influenced by nutrition information were also greater among participants trying to lose or maintain their weight without disordered eating, but not for participants trying to gain weight (Table 5).

Compared to the reference group, participants with binge eating had greater odds of ordering something different (OR = 4.51 [95% CI: 2.68 to 7.61]), eating less of the food ordered (OR = 6.53 [95% CI: 3.29 to 12.96]), eating out less often (OR = 3.76 [95% CI: 2.14 to 6.60]), and changing restaurant locations (OR = 8.56 [95% CI: 3.91 to 18.74]) (Table 4).

Participants with self-induced vomiting also had higher odds of ordering something different (OR = 7.52 [95% CI: 3.80 to 14.88]), eating less of the food ordered (OR = 8.83 [95% CI: 4.33 to 17.99]), eating out less often (OR = 4.48 [95% CI: 2.15 to 9.36]), and changing restaurant locations (OR = 18.65 [95% CI: 7.21 to 48.23]).

Similarly, participants preoccupied with thinness had greater odds of ordering something different (OR = 4.72 [95% CI: 2.96 to 7.53]), eating less of the food ordered (OR = 5.74 [95% CI: 2.90 to 11.36]), eating out less often (OR = 2.58 [95% CI: 1.55 to 4.33]), and changing restaurant locations (OR = 7.58 [95% CI: 3.56 to 16.12]).

Compared to the reference group, participants who reported trying to lose weight had 1.79 (95% CI:1.09 to 2.94) greater odds of having ordered something different (Table 5), but the odds did not differ for those trying to gain or maintain weight. There were no differences in the odds for having eaten less of the food ordered due to nutrition information in any of the three weight management groups. Odds of eating out less often were greater among participants trying to lose or maintain weight compared with the no disordered eating and no weight management reference group (ORs = 2.69 [95% CI: 1.61 to 5.42] and 2.83 [95% CI: 1.65 to 4.87], respectively). There were no differences in the odds for having changed restaurants due to nutrition information in any of the three weight management groups (Table 5).

Participants who reported a preoccupation with thinness had 2.01 (95% CI: 1.52 to 2.66) times the odds of supporting a calorie labelling policy compared to those with no disordered eating or weight management efforts (Table 4). Participants who reported self-induced vomiting were 0.66 (95% CI: 0.46 to 0.95) times less likely to support the policy compared to those not engaging in disordered eating or weight management behaviours. Participants who reported binge eating did not differ significantly in odds of policy support compared to the reference group. Participants who reported trying to lose weight and did not report any disordered eating had 1.97 (95% CI: 1.49 to 2.59) times the odds of supporting a calorie labelling policy compared to the reference group, but there were no significant differences in odds for those trying to gain or maintain weight compared to those not trying to manage their weight.

## Discussion

To our knowledge, this is one of the first quantitative studies in England to examine how adults with disordered eating and weight management efforts view and engage with a potential menu labelling policy and nutritional information in restaurants.

A calorie labelling policy for large food businesses (defined as businesses that serve food with 250 or more employees) in England was announced in July 2021 and implemented in April 2022 [12]. The policy was introduced as “Tackling obesity: empowering adults and children to live healthier lives” [45] to potentially benefit certain groups who use the information to make lower calorie choices. Just over 18% of participants who visited a restaurant in the last 6 months reported noticing any nutrition information at their last restaurant visit. A recent UK-based study reported similar rates (16.5%) of noticing labels prior to the mandatory calorie-labelling policy implementation in England [46]. Furthermore, a 2018 cross-sectional study of 100 popular fast-food restaurants in the UK found that just over 30% provided menu labels [47, 48], which may explain the lower rates of noticing nutrition information in our study compared to other studies outside of the UK [49]. The respondents in our study may have also been reporting noticing of nutrition information other than calorie labels on menus, for example, nutrition information on tray liners, nutrition pamphlets or napkins.

Recent evaluations of England’s calorie labelling policy showed an increase in self-reported noticing of calorie labels and ordering something different after the policy was fully implemented [50]. On the other hand, a customer intercept survey of approximately 3,300 participants did not find a significant change in calories purchased or consumed [46]. Nonetheless, this study may be a helpful baseline for future analyses now that the menu labelling policy has been implemented in England. This study provides baseline information for a real-world policy that was implemented after the survey data were collected, including on the relationship between disordered eating behaviours, weight management, and nutrition information and policy support for calorie labels.

Participants in all the disordered eating groups were more likely to have noticed nutrition information at their last restaurant visit compared to the reference group. There were no differences in noticing nutrition information between the any of the weight management groups and the reference group. This could suggest that the disordered eating group may have a heightened focus on food choices or nutritional content in restaurants, potentially due to concerns related to eating patterns or body image, or using labels as a tool to support weight modification or to enable disordered eating [32, 34].

Among the weight management groups, only participants trying to lose or maintain weight had greater odds of being influenced by the nutrition information compared to the reference group. This aligns with previous studies showing that adults trying to lose weight are more likely to report using nutritional information at fast food restaurants compared to those not trying to lose weight [51]. Our results regarding the disordered eating groups are similar to previous findings that using menu labels was associated with unhealthy weight-control behaviours and binge eating behaviours in women [36, 49].

All three disordered eating groups, and the weight loss and weight maintenance groups were more likely to report eating out less often because of nutrition information compared to the reference group. This is a notable finding, as consumers typically only see such information once they are already at the restaurant. The disordered eating groups were also more likely to report changing restaurants due to nutrition labels, suggesting possible avoidance of establishments with nutritional labelling, potentially driven by heightened awareness of these labels, although this was not explored in our analyses.

Additionally, the disordered eating groups were more likely to report ordering something different and eating less of what they ordered because of nutrition information. In contrast, only the weight loss group had higher odds of ordering something different and did not show increased odds for the other behaviours compared to the reference group. This further suggests that individuals with disordered eating may have a heightened focus on food choices and nutritional content in restaurant settings compared to those without disordered eating.

Previous work suggests that the impact of nutrition information among individuals with eating disorders is heterogeneous. A systematic review identified that some individuals experience nutrition labels as providing safety and reassurance, by offering a sense of predictability and control over food choices [32]. In contrast, there are concerns that nutrition information may reinforce unhealthy behaviours, such as increasing attention on calories or avoiding eating in social settings, among people experiencing disordered eating [32].

Over half (56.9%) of all participants reported supporting a government policy requiring calorie amounts on chain restaurant menus (Table 3), consistent with previous findings that people generally support calorie labelling practices, regardless of disordered eating, weight, or dieting status [52].

We found that the preoccupation with thinness group leaned towards greater policy support compared to the reference group, whereas participants who reported self-induced vomiting, in contrast, had decreased odds of policy support and there was no significant difference in support among participants who reported binge eating. The relationship between disordered eating and policy support is complex. Previous studies have reported that people may source food for binges from restaurants, and that eating at restaurants is perceived as uncontrolled and excessive more often by women who engage in binge eating [53]. Qualitative work has found that people with lived experience of eating disorders reported increased distress and avoidance of eating out after calorie labels were introduced [35] and have highlighted feelings of shame, guilt, and tension due to calorie labels [33–35]. Our findings showed that there is evidence that some people with disordered eating support calorie labels on menus, but this varied by type of disordered eating behaviour. It is unclear whether this support was driven by the intention to use the labels as a weight-loss tool or to engage in unhealthy behaviours, or whether it reflects other motivations, such as increasing awareness and autonomy over caloric intake, much like the general public [33–35].

### Strengths and limitations

A key strength of this study included the large sample size. Our sample included a high prevalence of participants reporting individual disordered eating behaviours, which is comparable to other studies [26, 27, 29]. However, the disordered eating measures used in the current study are not inclusive of all disordered eating behaviours and attitudes, so there could still be participants in the weight management groups engaging in disordered eating other than the behaviours assessed in this study (e.g., over-exercise, severe caloric restriction). This study separated those who are managing their weight from those with disordered eating, which reduced heterogeneity within the weight management group to a greater extent than prior work. By including participants who were trying to gain, lose, or maintain weight, we captured a wider range of participants who may engage with nutritional information in a variety of ways.

This study also has some limitations. Measures of noticing, influence, and impact referred broadly to “nutrition information,” which may have included sources other than calorie labels on restaurant menus, limiting conclusions about calorie labelling specifically. In addition, the menu labelling policy had not yet been implemented at the time of data collection (and would only have applied to chain restaurants), potentially reducing opportunities for exposure; noticing of this information may have increased once the labels became mandatory in chain restaurants in April 2022. Despite the reduced chances of encountering nutritional labels in restaurants, our findings show clear differences in impact and influence of labels for people living with disordered eating and weight management efforts.

As the study is cross-sectional and based on a single pre-implementation survey, no temporal or causal relationships can be inferred between disordered eating, nutrition label use, and policy support. It remains unclear whether nutrition labels contribute to unhealthy behaviours among people with disordered eating or are instead sought out to support pre-existing behaviours. The use of self-reported measures is another limitation of this study. Participants may feel reluctant to share how often or if they engage in unhealthy weight-loss methods, and our disordered eating measures may be underreported; however, this is likely somewhat alleviated by the anonymity of online surveys. Additionally, our outcome measures are reliant on participants’ memories and could reduce the accuracy of these measures.

## Conclusion

Overall, this study describes the prevalence of noticing and using nutrition information in restaurants and public support for calorie labels on menus among adults in England engaged in disordered eating, attempting to manage their weight, or neither, prior to the implementation of calorie labelling in England’s out-of-home food sector. Participants living with disordered eating were more likely to notice and be influenced by nutrition labels, and there were variations in support by disordered eating and weight management behaviours.

Further research is needed to examine in greater detail how people living with disordered eating engage with the mandatory menu labelling policy and to examine whether policy support, noticing nutrition information, and behaviours changed after policy implementation in England. As the current study has identified ways in which those with disordered eating engage differently with nutrition information on menus, further research is warranted to ensure the policy is not causing undue harm among populations with and at risk of developing disordered eating.

## Supplementary Information

Supplementary Material 1.

## Data Availability

IFPS data is available upon reasonable request.
