# Supplementary material for: Examining use of restaurant nutrition information among adults living in England engaged in disordered eating or weight management efforts
Source: BMC Public Health. 2026 Mar 31;26:1521. doi: 10.1186/s12889-026-27141-6 (PMC13162486; doi:10.1186/s12889-026-27141-6)
Supplement: Supplementary file 1 — Supplementary Material 1. [file 12889_2026_27141_MOESM1_ESM.docx]

**Appendix**

*Supplementary Table 1:* *Sociodemographic Classifications*

| ***Variable*** | ***Question*** | ***Response Options/ Coding*** |
| --- | --- | --- |
| Sex | What sex were you assigned at birth, meaning on your original birth certificate? | 0= Male  1= Female |
| Gender | What is your current gender identity? | 1=Man  2=Woman  3=Trans male/trans man  4=Trans female/trans woman 5=Gender queer/gender nonconforming  6=Different identity  -99=Not stated |
| Ethnicity | Which of the following best describes your ethnic or racial background? | 1=White (including Gypsy/Traveller/Irish Traveller)  2=Mixed/Multiple Ethnic Groups  3=Asian/Asian British  4=Black/African/Caribbean/ Black British  5=Other Ethnic Group  -99=Not stated (recoded as missing) |
| Education | What is the highest level of formal education that you have completed? | 0= Secondary school level or less: GCSEs, 1+ A levels, Apprenticeship  1= Postsecondary school or higher: Degree, Higher Degree, Professional qualifications (for example teaching, nursing, accountancy) |
| Income adequacy | “Thinking about your total monthly income, how difficult or easy is it for you to make ends meet?” | 0= “Not easy”: very difficult, difficult, neither difficult or easy  1= “Easy”: easy, very easy |

*Supplementary Table 2: E*xposure Group Classifications

| ***Exposure Group*** | ***Question*** | ***Response Options*** | ***Coding*** |
| --- | --- | --- | --- |
| ***Reference group  (No weight management or disordered eating)*** | NA | NA | Did not meet any of the EAT-3 criteria and did not select any weight management items |
| ***Binge eating*** | In the past 3 months, how often have you: … gone on eating binges? (Eating a large amount of food while feeling out of control). | 1=Never  2=Less than 1 time a month  3=1 to 3 times a month  4=Once a week  5=2 to 6 times a week  6=Once a day 7=More than once a day  -77=Don’t know  -88=Refuse to answer | Yes  (=3-7) |
| ***Self-induced vomiting*** | In the past 3 months, how often have you: … made yourself sick (vomited) to control your weight? | 1=Never  2=Less than 1 time a month  3=1 to 3 times a month  4=Once a week  5=2 to 6 times a week  6=Once a day 7=More than once a day  -77=Don’t know  -88=Refuse to answer | Yes  (=2-7) |
| ***Preoccupation with thinness*** | I am preoccupied with a desire to be thinner | 1=Always  2=Usually  3=Often 4=Sometimes 5=Rarely  6=Never  -77=Don’t know  -88=Refuse to answer | Yes  (=1-3) |
| ***Weight loss efforts without disordered eating*** | During the past 12 months have you tried to….  Lose weight? | 0=Unchecked 1=Checked | Yes (1)  (Checked the weight loss item and did not meet cut point for any of the EAT-3 items) |
| ***Weight gain efforts without disordered eating*** | During the past 12 months have you tried to….  Gain weight? | 0=Unchecked 1=Checked | Yes (1)  (Checked the weight gain item and did not meet cut point for any of the EAT-3 items) |
| ***Weight maintenance efforts without disordered eating*** | During the past 12 months have you tried to….  Stay the same weight? | 0=Unchecked 1=Checked | Yes (1)  (Checked the weight maintenance item and did not meet cut point for any of the EAT-3 items) |

Supplementary Table 3 Prevalence of each exposure group in study sample (weighted n (%)) (n=2,898)

| **Group** | **Weighted n (weighted % of total n)** |
| --- | --- |
| Binge eating | 779 (26.9) |
| Vomit behaviour | 530 (18.3) |
| Preoccupied with thinness | 832 (28.7) |
| Weight gain efforts, without DE | 83 (2.9) |
| Weight loss efforts, without DE | 581 (20.1) |
| Weight maintenance, without DE | 387 (13.4) |
| Reference group (no disordered eating and no weight management) | 624 (21.5) |

Supplementary Table 4 Demographic Characteristics of the Exposure groups (weighted n; (n%))

|  | *Binge (n=779)* | *Vomit*  *(n=530)* | *Thin*  *(n=832)* | *Loss*  *(n=581)* | *Gain*  *(n=83)* | *Same*  *(n=387)* |
| --- | --- | --- | --- | --- | --- | --- |
| *Female* | *389(49.9%)* | *239(45.1%)* | *488*  *(58.7%)* | *321*  *(55.25)* | *32*  *(38.6%)* | *173*  *(44.7%)* |
| *White* | *610(78.3%)* | *398(75.0%)* | *671(80.6%)* | *547(94.1%)* | *54(65.1%)* | *348(89.9%)* |
| *Mixed/Other* | *74(9.5%)* | *68(12.8%)* | *72(8.7%)* | *4 (0.7%)* | *9(10.8%)* | *11 (2.8%)* |
| *Black* | *70(9.0%)* | *43(8.1%)* | *72(8.7%)* | *14 (2.4%)* | *12(14.5%)* | *20(5.2%)* |
| *Asian* | *25(3.2%)* | *20(3.8%)* | *16(1.9%)* | *15(2.6%)* | *7(8.4%)* | *9(2.3%)* |
| *High Education* | *252(32.3%)* | *198(37.4%)* | *264(31.7%)* | *163 (28.1%)* | *20(24.1%)* | *135(34.9%)* |
| *High income adequacy* | *263(33.8%)* | *181 (34.2)* | *291(35.0%)* | *278(47.8%)* | *31(37.3%)* | *221 (57.1%)* |

*Supplementary Table 5 Frequencies* of disordered eating behaviour by weight management behaviour; unweighted n (adjusted weighted %) (n=2,883)*

|  | No weight management | Any weight management |
| --- | --- | --- |
| No disordered eating | 631 (21.7%) | 1057 (35.0%) |
| Disordered eating  (Met cut off for at least one disordered eating item) | 120 (4.2%) | 1075 (37.3%) |

**15 participants have missing responses for the EAT-3 items*
